# Distal Tibial Oblique Osteotomy Combined with Strut Bone Allografting for the Treatment of Ankle Osteoarthritis with Varus Deformity: A Case Series

**DOI:** 10.3390/jcm15166298

**Published:** 2026-08-14

**Authors:** Meng-Chun Tsai, Yi-Hsuan Lee, Tung-Ying Lee, Yi-Chen Li, Kai-Chiang Yang, Chen-Chie Wang

**Affiliations:** 1 Department of Orthopedic Surgery, Taipei Tzu Chi Hospital, Buddhist Tzu Chi Medical Foundation, New Taipei City 231016, Taiwan; tch36281@tzuchi.com.tw (M.-C.T.); tch27228@tzuchi.com.tw (Y.-H.L.); tch34095@tzuchi.com.tw (T.-Y.L.); momoqsa@tzuchi.com.tw (Y.-C.L.); pumpkin@tmu.edu.tw (K.-C.Y.); 2 School and Graduate Institute of Physical Therapy, College of Medicine, National Taiwan University, Taipei 100233, Taiwan; 3 Graduate Institute of Biomedical Materials and Tissue Engineering, College of Biomedical Engineering, Taipei Medical University, Taipei 110301, Taiwan; 4 School of Dental Technology, College of Oral Medicine, Taipei Medical University, Taipei 110301, Taiwan; 5 Department of Orthopedics, School of Medicine, Tzu Chi University, Hualien 970473, Taiwan

**Keywords:** ankle osteoarthritis, varus deformity, distal tibial oblique osteotomy, alignment correction, strut bone allograft

## Abstract

**Background:** Advanced varus ankle osteoarthritis is challenging because correction of malalignment must be balanced against preservation of the native ankle joint. This study evaluated the clinical and radiographic outcomes of distal tibial oblique osteotomy (DTOO) with structural strut allografting, which permits individualized correction and provides mechanical support to the osteotomy gap. **Methods:** This retrospective case series included 20 patients and 20 operated ankles with Takakura stage IIIa (*n* = 13) or IIIb (*n* = 7) varus ankle osteoarthritis who underwent DTOO with structural strut allografting between 2012 and 2024. Clinical outcomes included Manchester–Oxford Foot Questionnaire (MOXFQ), American Orthopaedic Foot & Ankle Society (AOFAS), 12-Item Short-Form Survey (SF-12), and Visual Analog Scale (VAS) pain scores. Radiographic parameters included tibial articular surface angle (TAS), tibial lateral surface angle (TLS), medial malleolar angle (MMA), talar tilt angle (TTA), and tibiotalar surface angle (TTS). **Results:** Significant improvements were observed at final follow-up. MOXFQ scores decreased from 57.66 ± 22.01 to 10.63 ± 9.72 (*p* < 0.001), AOFAS scores increased from 63.75 ± 15.17 to 85.45 ± 8.37 (*p* < 0.001), and VAS pain scores decreased from 4.30 ± 1.75 to 1.00 ± 1.30 (*p* < 0.001). The SF-12 PCS increased from 45.23 ± 8.48 to 47.96 ± 7.93 (*p* = 0.258), and the MCS increased from 53.51 ± 9.85 to 55.60 ± 4.75 (*p* = 0.377); neither change was statistically significant. Significant correction was achieved in TAS (*p* < 0.001), TLS (*p* < 0.001), MMA (*p* = 0.019), and TTS (*p* < 0.001), with no significant change in TTA (*p* = 0.336). All achieved radiographic union within 3 months postoperatively. **Conclusions:** DTOO with strut bone allografting enables alignment correction in advanced varus ankle osteoarthritis, including Takakura stage III, while preserving the ankle joint. This technique is an effective joint-preserving option for severe deformities.

## 1. Introduction

Ankle osteoarthritis is a progressive and debilitating condition characterized by degeneration of articular cartilage, leading to pain, stiffness, and functional impairment [1]. Ankle-specific epidemiological data remain limited because major global burden analyses do not report ankle OA as a separate category. Osteoarthritis overall affected an estimated 595 million people worldwide in 2020 [2]. In a population-based study from the United Kingdom, the prevalence of symptomatic radiographic ankle OA among community-dwelling adults aged 50 years or older was 3.4% (95% confidence interval, 2.3–4.5%) [3]. Varus ankle osteoarthritis represents a distinct mechanical pattern in which cartilage degeneration is accompanied by distal tibial malalignment, asymmetric medial joint-space narrowing, and medial displacement of load across the tibiotalar joint [4]. Therefore, treatment must address not only articular degeneration but also the underlying deformity and loss of joint congruity. Realignment procedures, such as supramalleolar osteotomy (SMO), are designed to restore alignment and alleviate pain; however, these methods may be less suitable in advanced cases or in cases involving severe varus deformities [5].

Various classification systems have been developed to evaluate the severity and stage of ankle osteoarthritis. These systems support treatment decision-making and outcome prediction by categorizing the extent of joint degeneration and deformity. For example, in the Takakura–Tanaka classification system, stage I indicates mild degeneration, whereas stage IV indicates severe joint destruction and deformity. Identifying the stage of osteoarthritis is crucial for selecting the appropriate surgical intervention and setting realistic expectations for recovery [6].

In conventional practice, early- and mid-stage ankle osteoarthritis, classified as stages I and II, is typically managed with arthroscopic debridement or joint distraction techniques [7]. In advanced-stage ankle osteoarthritis, classified as stages III and IV, more invasive interventions, such as ankle fusion (arthrodesis) or total ankle replacement, are often required [8]. Notably, these 2 techniques have certain limitations. Ankle arthrodesis can provide reliable pain relief and create a stable, plantigrade ankle in patients with advanced varus deformity; however, it eliminates tibiotalar motion and may increase mechanical loading of adjacent joints [9]. Total ankle replacement preserves ankle motion and may improve function in appropriately selected patients, but its use requires adequate bone stock, soft-tissue balance, and correctable alignment, and concerns remain regarding implant durability and subsequent revision [10]. These trade-offs highlight the need for joint-preserving procedures such as distal tibial oblique osteotomy (DTOO) in carefully selected patients with advanced but potentially salvageable ankle joints. For patients with mid-stage disease or selected cases of advanced-stage disease, joint-preserving osteotomy remains a feasible option.

Among joint-preserving realignment procedures, conventional SMO is effective for coronal correction; however, because the osteotomy plane is often located proximal to the center of rotation and angulation, correction of a substantial varus deformity may result in unintended lateral translation of the distal tibial fragment [11]. DTOO directs the osteotomy obliquely toward the distal tibiofibular syndesmosis, creating a pivot closer to the ankle joint. This configuration may facilitate substantial angular and rotational correction while limiting translational displacement of the distal fragment. Although DTOO has previously been performed using β-tricalcium phosphate or autograft [12], our technique uses an individually contoured structural strut allograft to accommodate the osteotomy gap, permit adjustment of the correction, and provide immediate mechanical support. These characteristics may be particularly useful in selected patients with advanced but correctable varus deformity requiring substantial correction.

Previous studies have reported favorable clinical and radiographic outcomes after DTOO using β-tricalcium phosphate or autologous bone grafting [12]. However, evidence regarding DTOO combined with structural strut allografting in patients with Takakura stage IIIa and IIIb varus ankle osteoarthritis remains limited. The present technique uses an individually contoured structural strut allograft to accommodate the osteotomy gap, provide immediate mechanical support, and avoid morbidity associated with autologous graft harvesting. In this study, we evaluated the functional, pain-related, and radiological outcomes associated with DTOO combined with strut bone allografting in selected patients with advanced varus ankle osteoarthritis. We hypothesized that this procedure would improve patient-reported outcomes, correct radiographic alignment, and achieve reliable bone union in patients with Takakura stage IIIa and IIIb disease.

## 2. Materials and Methods

### 2.1. Study Design and Patients

This retrospective consecutive case series was approved by the Institutional Review Board of Taipei Tzu Chi Hospital, Buddhist Tzu Chi Medical Foundation (approval number: 13-IRB122; approval date: 19 December 2024) and was conducted in accordance with the Declaration of Helsinki. The requirement for informed consent was waived because of the retrospective nature of the study and the use of anonymized data.

Patients were identified by screening the institutional operative lists for DTOO procedures performed between September 2012 and February 2024. All patients who met the predefined eligibility criteria were consecutively included. The cohort comprised 20 patients and 20 operated ankles, with no bilateral procedures; the operated ankle was the unit of analysis. In addition to the predefined eligibility criteria, final candidacy for DTOO was determined by the treating surgeon based on preservation of the lateral tibiotalar cartilage, correctability of the deformity, and the potential to restore tibiotalar joint congruity.

The inclusion criteria were as follows: (1) ankle osteoarthritis with varus deformity classified as Takakura stage IIIa or IIIb; (2) treatment with DTOO combined with structural strut bone allografting; (3) availability of preoperative and postoperative weight-bearing ankle radiographs; and (4) a minimum follow-up duration of 24 months. Patients were excluded if they had infectious arthritis, congenital deformity involving the lower limb or ankle, neuroarthropathy, incomplete clinical or radiographic data, or loss to follow-up before 24 months. Diabetes mellitus and hypertension were not exclusion criteria and were not systematically analyzed as independent study variables. All patients underwent preoperative clinical evaluation of the ipsilateral hip and knee and overall lower-limb alignment. Clinically significant hip or knee pathology or deformity was treated before ankle reconstruction. Therefore, the included patients had no untreated proximal-joint deformity considered likely to substantially affect lower-limb alignment or ankle symptoms.

### 2.2. Surgical Technique

All patients were placed in the supine position and underwent surgery under either general or spinal anesthesia. A pneumatic tourniquet was applied to the ipsilateral thigh throughout the procedure.

Standard anteromedial and anterolateral portals were established for ankle arthroscopy. Initial diagnostic arthroscopy was performed using a 4.0 mm 30° arthroscope (Stryker Endoscopy, San Jose, CA, USA) to evaluate the extent and distribution of chondral degeneration and subchondral bone exposure in the tibiotalar joint. Unstable or degenerated cartilage was debrided, and microfracture was performed where indicated to stimulate fibrocartilaginous repair. Comprehensive arthroscopic debridement of impinging anterior, anterolateral, and lateral gutter osteophytes, as well as loose bodies, was performed to restore talar mobility and facilitate correction. Medial gutter osteophytes were additionally removed when they contributed to impingement or restricted joint motion.

**Figure 1:**
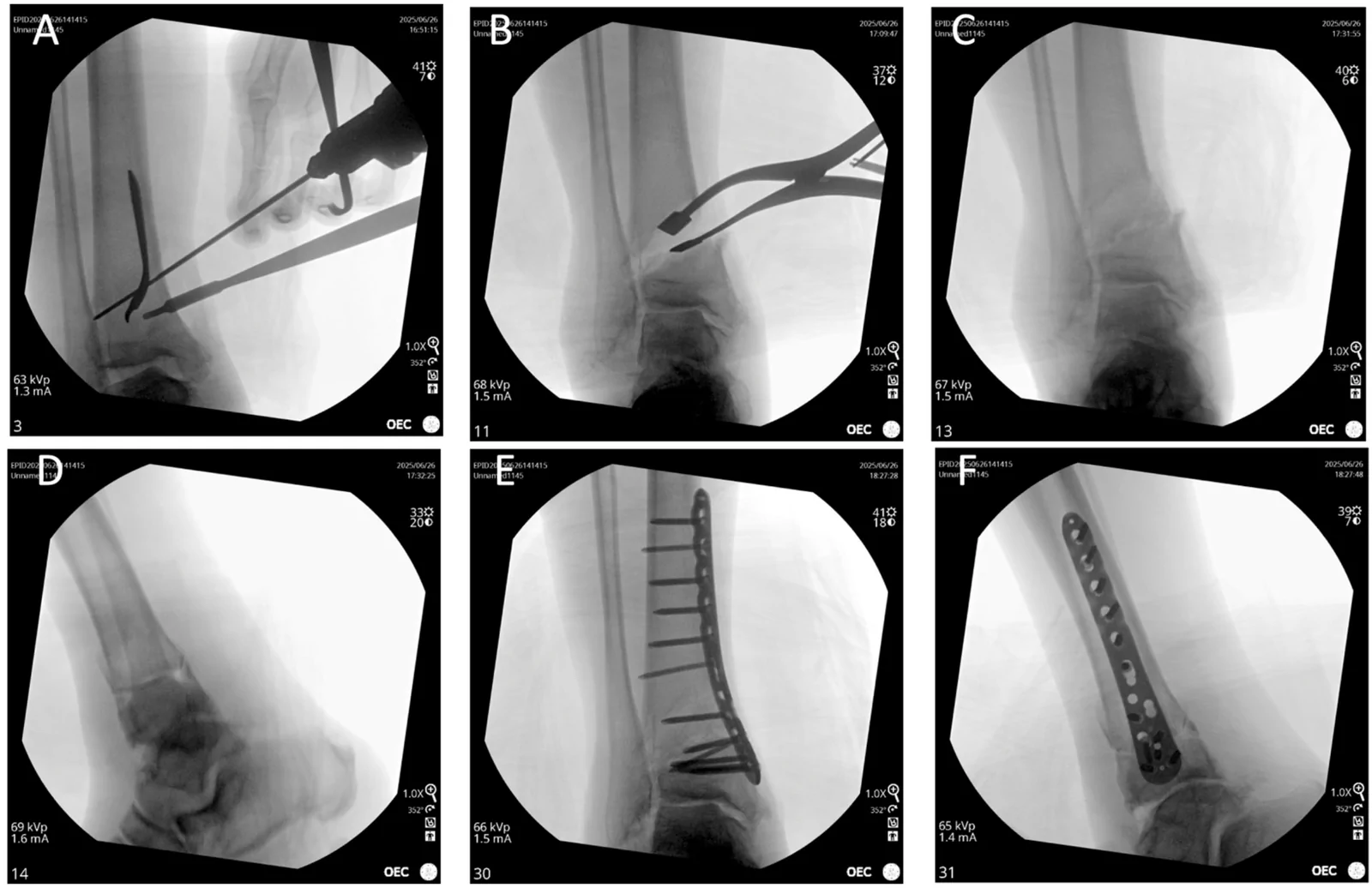
Intraoperative fluoroscopic images. (**A**) Initial oblique osteotomy was performed from the medial tibial cortex under C-arm guidance. (**B**) A lamina spreader was inserted to gradually open the osteotomy site, preparing the gap for strut allograft insertion. (**C**) Fluoroscopic confirmation of the correction angle and medial gap size was performed after allografting. (**D**) Lateral view demonstrating preserved ankle joint alignment. (**E**) Fixation was achieved using a medial distal tibial locking plate with multiple screws crossing the osteotomy and allograft site. (**F**) Final lateral image confirming proper implant position, angular correction, and graft support.

The oblique osteotomy line was planned to begin approximately 5 cm proximally to the tip of the medial malleolus and to extend laterally toward the center of the distal tibiofibular syndesmosis. A medial longitudinal skin incision measuring approximately 3 cm was made over the distal tibia at the planned osteotomy entry point. Three 2.0 mm Kirschner wires were inserted parallel to each other along the medial surface of the distal tibia, with a proximal–medial to distal–lateral trajectory toward the upper point of the syndesmosis. An osteotomy was performed along the guide wires using a powered saw. A laminar spreader was inserted into the osteotomy gap, and gradual opening was performed under fluoroscopic guidance (GE OEC Medical Systems, Salt Lake City, UT, USA). The osteotomy gap was enlarged under fluoroscopic guidance until slight overcorrection of the varus deformity to approximately 4–5° of valgus alignment was achieved. This target was selected based on the senior surgeon’s experience and published recommendations for slight valgus overcorrection to avoid residual varus alignment and redistribute loading away from the medial tibiotalar compartment [11,13]. Because no consensus has been established regarding the optimal correction angle, the 4–5° target was used as an intraoperative guide rather than as a rigid radiographic threshold [14]. Neither fibular osteotomy nor Achilles tendon lengthening was required in any patient (Figure 1).

At our institution, an established bone bank supported by body donation provides a reliable supply of fresh-frozen structural allografts. The grafts were handled according to standard institutional bone-bank protocols and were not subjected to additional irradiation, chemical sterilization, or other terminal sterilization. This allows anatomically matched structural grafts from the corresponding skeletal site to be routinely available for medial opening-wedge DTOO. Consequently, structural allografts have become our preferred grafting material because they provide immediate structural support, avoid donor-site morbidity associated with autologous iliac crest bone graft harvesting, and can be individually contoured to match the osteotomy gap and correction angle.

**Figure 2:**
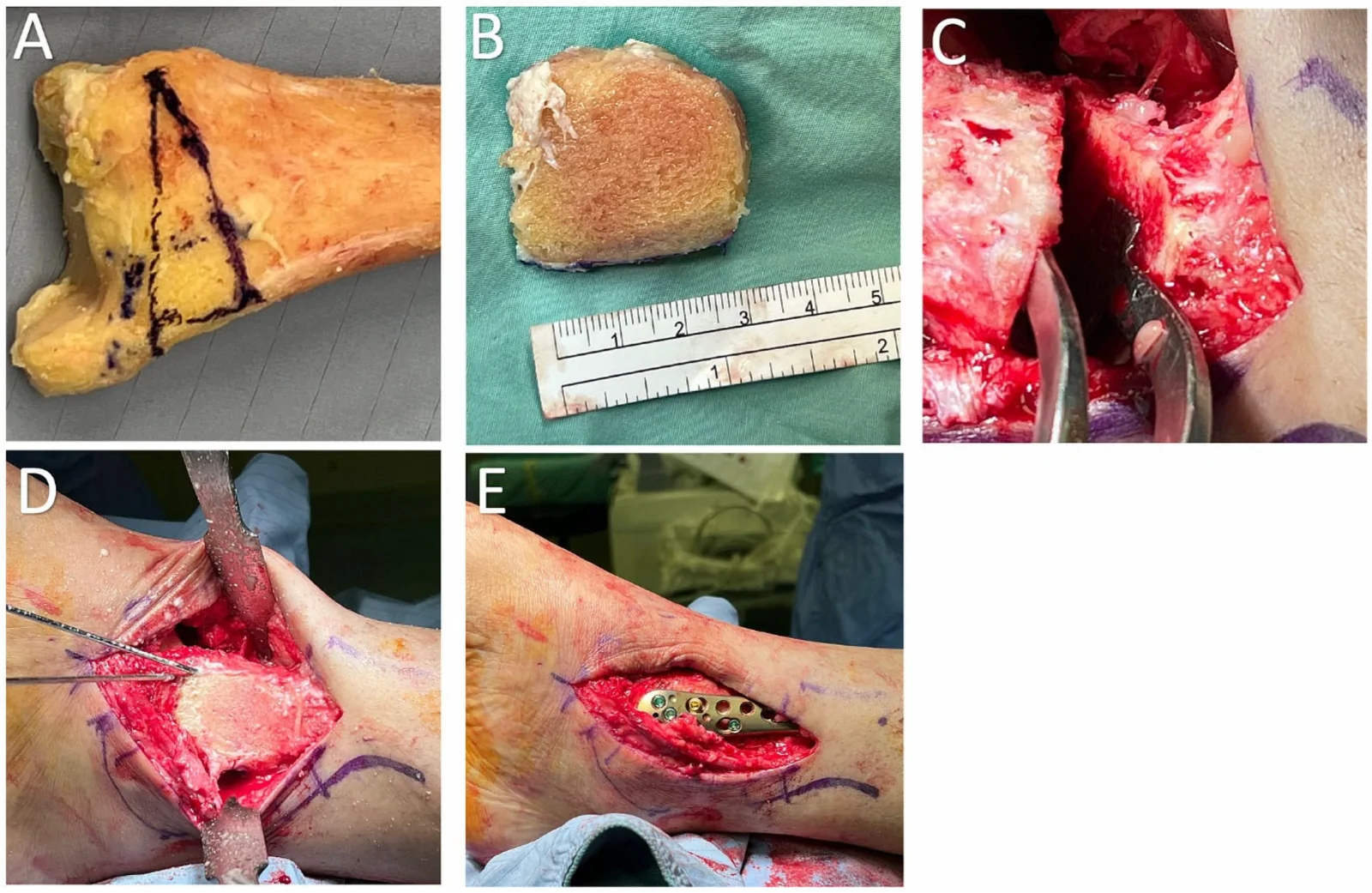
Strut allograft preparation and surgical implantation. (**A**) A fresh-frozen allograft harvested from the same anatomic region is being contoured to match the patient’s medial wedge-shaped osteotomy site. The anterior–posterior height is asymmetrically shaped to accommodate rotational correction and volumetric reduction. (**B**) The trimmed strut allograft measured approximately 3 cm in height, designed to restore medial support and maintain angular correction. (**C**) Intraoperative view showing the open osteotomy site with cancellous bone exposed after distraction. (**D**) The prepared strut allograft was carefully impacted into the medial gap under compression and temporarily fixed with multiple Kirschner wires. (**E**) A medial locking plate was then applied to secure both the distal tibial segment and the strut graft, completing fixation.

Graft dimensions were not standardized; instead, each structural strut allograft wedge block was individually contoured to conform to the medial opening-wedge osteotomy site and planned correction. The anterior–posterior height of the graft was adjusted when necessary to accommodate rotational correction and improve contact with the osteotomy surfaces. The prepared allograft was implanted into the osteotomy gap under compression. After the correction position was confirmed fluoroscopically, fixation was achieved using either a locking plate or a conventional plate. The uneven medial tibial cortex and cancellous bone were trimmed when necessary to facilitate secure plate application and allow wound closure without excessive skin tension. A drain was placed at the osteotomy site, and the limb was immobilized in a splint after wound closure (Figure 2).

After completion of osteotomy correction and fixation, lateral ankle stability was reassessed using the anterior drawer and talar tilt tests under fluoroscopic guidance, together with arthroscopic evaluation of the lateral ligament complex. Concomitant anterior talofibular ligament (ATFL) repair was performed when persistent lateral ankle instability was identified after bony correction. In patients without residual instability after realignment, no ligament repair was performed.

### 2.3. Postoperative Protocol

Postoperatively, the affected limb was immobilized in a splint during the early healing period. Partial weight bearing was permitted for between 4 and 6 weeks after surgery according to radiographic progression and clinical tolerance. Weight bearing was then gradually increased on a weekly basis, with full weight bearing expected at approximately 3 months postoperatively.

### 2.4. Clinical Outcome Assessment

Clinical outcomes were assessed using the Manchester–Oxford Foot Questionnaire (MOXFQ), American Orthopaedic Foot & Ankle Society (AOFAS) ankle-hindfoot score, 12-Item Short-Form Survey (SF-12), and Visual Analog Scale (VAS) pain score. The SF-12 was reported using the Physical Component Summary (PCS) and Mental Component Summary (MCS) scores. These are norm-based component scores, with higher values indicating better physical or mental health. No combined SF-12 total score was calculated. Preoperative MOXFQ, AOFAS, SF-12, and VAS pain scores were obtained before surgery, and postoperative scores were assessed at the final follow-up visit.

### 2.5. Radiographic Assessment and Bone Union

Weight-bearing anteroposterior and lateral ankle radiographs were obtained preoperatively and during postoperative follow-up. Full-length weight-bearing lower-limb radiographs were not routinely obtained; therefore, limb-length discrepancy and global lower-limb mechanical-axis deformity were not quantitatively included in the radiographic analysis. Radiographic assessment included the tibial articular surface angle (TAS), tibial lateral surface angle (TLS), medial malleolar angle (MMA), talar tilt angle (TTA), and tibiotalar surface angle (TTS). Measurements were performed on standardized weight-bearing radiographs according to previously described methods. All radiographic measurements were performed by the first author (M.-C.T.). To minimize measurement error, each radiographic parameter was measured twice during the same evaluation session, and the mean value of the two measurements was used for statistical analysis.

Bone union was adjudicated using serial plain radiographs together with clinical assessment. Radiographic criteria included bridging trabecular continuity across the osteotomy and host–allograft interface without progressive radiolucency or implant loosening, whereas the clinical criterion was the absence of pain at the osteotomy site during weight bearing. Maintenance of correction was evaluated by comparing postoperative alignment parameters with those at the final follow-up.

### 2.6. Complications and Secondary Procedures

Postoperative complications and secondary procedures were reviewed using medical records and serial radiographs. Major complications were defined as deep surgical-site infection, nonunion, graft collapse, loss of correction, revision osteotomy, revision grafting, conversion to ankle arthrodesis, or conversion to total ankle arthroplasty. Secondary procedures, including implant removal after confirmed union because of symptomatic hardware irritation or foreign-body sensation, were recorded separately.

### 2.7. Statistical Analysis

Statistical analyses were performed using IBM SPSS Statistics for Windows, version 27.0 (IBM Corp., Armonk, NY, USA). Continuous variables are presented as means and standard deviations. Preoperative and postoperative radiographic parameters were compared using paired *t* tests. Patient-reported outcome measures were analyzed using the Wilcoxon signed-rank test to account for nonnormal distribution and potential floor effects. A *p* value of <0.05 was considered statistically significant.

Exploratory subgroup analyses were performed according to preoperative Takakura stage (IIIa vs. IIIb). Within each subgroup, preoperative and postoperative patient-reported outcomes were compared using the Wilcoxon signed-rank test, and radiographic parameters were compared using paired *t* tests. Between-group comparisons of baseline values, postoperative values, and change scores were performed using the Mann–Whitney U test. The change score was defined as the postoperative value minus the preoperative value. No adjustment for multiple comparisons was applied because the subgroup analysis was exploratory. Therefore, the subgroup findings were interpreted descriptively rather than as evidence of comparative efficacy between stages.

## 3. Results

### 3.1. Patient Characteristics and Follow-Up

The cohort comprised 20 patients and 20 operated ankles, including 13 right and 7 left ankles. According to the Takakura–Tanaka classification system, 13 ankles were categorized as stage IIIa and 7 as stage IIIb. The mean BMI was 27.15 ± 3.96 kg/m^2^ (range, 20.5–35.2 kg/m^2^). The mean follow-up duration was 51.4 months, with a range of 24 to 146 months (Table 1). The mean preoperative radiographic parameters were 83.13° ± 4.88° for TAS, 77.76° ± 5.60° for TLS, 35.93° ± 7.31° for MMA, 5.80° ± 6.70° for TTA, and 78.22° ± 6.87° for TTS. Concomitant ATFL repair was performed in 15 of the 20 patients because residual lateral ankle instability remained after correction of the osseous deformity.

### 3.2. Clinical Outcomes

Significant improvements in clinical outcomes were observed after surgery. As presented in Table 2, the MOXFQ scores decreased from 57.66 ± 22.01 preoperatively to 10.63 ± 9.72 postoperatively (*p* < 0.001), the AOFAS scores increased from 63.75 ± 15.17 preoperatively to 85.45 ± 8.37 postoperatively (*p* < 0.001), and the VAS pain scores decreased from 4.30 ± 1.75 preoperatively to 1.00 ± 1.30 postoperatively (*p* < 0.001). The SF-12 scores increased from 98.74 ± 11.37 preoperatively to 103.56 ± 7.64 postoperatively, although this difference was not statistically significant (*p* = 0.137).

### 3.3. Radiographic Outcomes

Radiographic assessments revealed significant improvements in alignment parameters after surgery. Specifically, the TAS increased from 83.13° ± 4.88° preoperatively to 92.88° ± 4.34° postoperatively (*p* < 0.001), the TLS increased from 77.76° ± 5.60° preoperatively to 82.94° ± 8.01° postoperatively (*p* < 0.001), the MMA decreased from 35.93° ± 7.31° preoperatively to 30.05° ± 8.36° postoperatively (*p* = 0.019), and the TTS increased from 78.22° ± 6.87° preoperatively to 87.79° ± 8.51° postoperatively (*p* < 0.001). The TTA decreased from 5.80° ± 6.70° preoperatively to 4.29° ± 3.68° postoperatively, but this change was not statistically significant (*p* = 0.336). These changes indicated improvement in coronal alignment and tibiotalar joint congruity after DTOO combined with strut bone allografting (Table 3).

### 3.4. Exploratory Subgroup Analysis by Takakura Stage

Subgroup analysis of patients with stage IIIa (*n* = 13) and stage IIIb (*n* = 7) disease revealed no significant between-group differences in preoperative or postoperative MOXFQ domain index scores, including pain, walking or standing, social interaction, or the MOXFQ total index. Similarly, no significant between-group differences were observed in the AOFAS total score, VAS pain score, or SF-12 physical and mental component summary scores before or after surgery (Table 4).

Regarding radiographic parameters, subgroup comparisons are summarized in Table 4. After surgery, no significant between-group differences were observed in postoperative TAS, TLS, MMA, TTA, or TTS. Patients with stage IIIb disease demonstrated a greater magnitude of TTA correction than those with stage IIIa disease (*p* = 0.024); however, postoperative patient-reported outcomes did not differ significantly between the 2 groups. Given the limited subgroup sizes and the absence of adjustment for multiple comparisons, this isolated significant finding and the overall subgroup results should be interpreted cautiously and descriptively.

### 3.5. Bone Union, Complications, and Secondary Procedures

At the final follow-up, radiographic bone union between the host bone and the allograft was observed in all patients. All patients achieved radiographic bone union within 3 months postoperatively. No major postoperative complications were observed during follow-up. Specifically, no cases of deep surgical-site infection, nonunion, graft collapse, loss of correction, revision grafting, conversion to ankle arthrodesis, or conversion to total ankle arthroplasty were observed during follow-up. Two patients underwent elective implant removal after confirmed union because of symptomatic hardware irritation or foreign-body sensation. These implant removals were classified as secondary procedures rather than major complications or treatment failures.

### 3.6. Representative Radiographs

**Figure 3:**
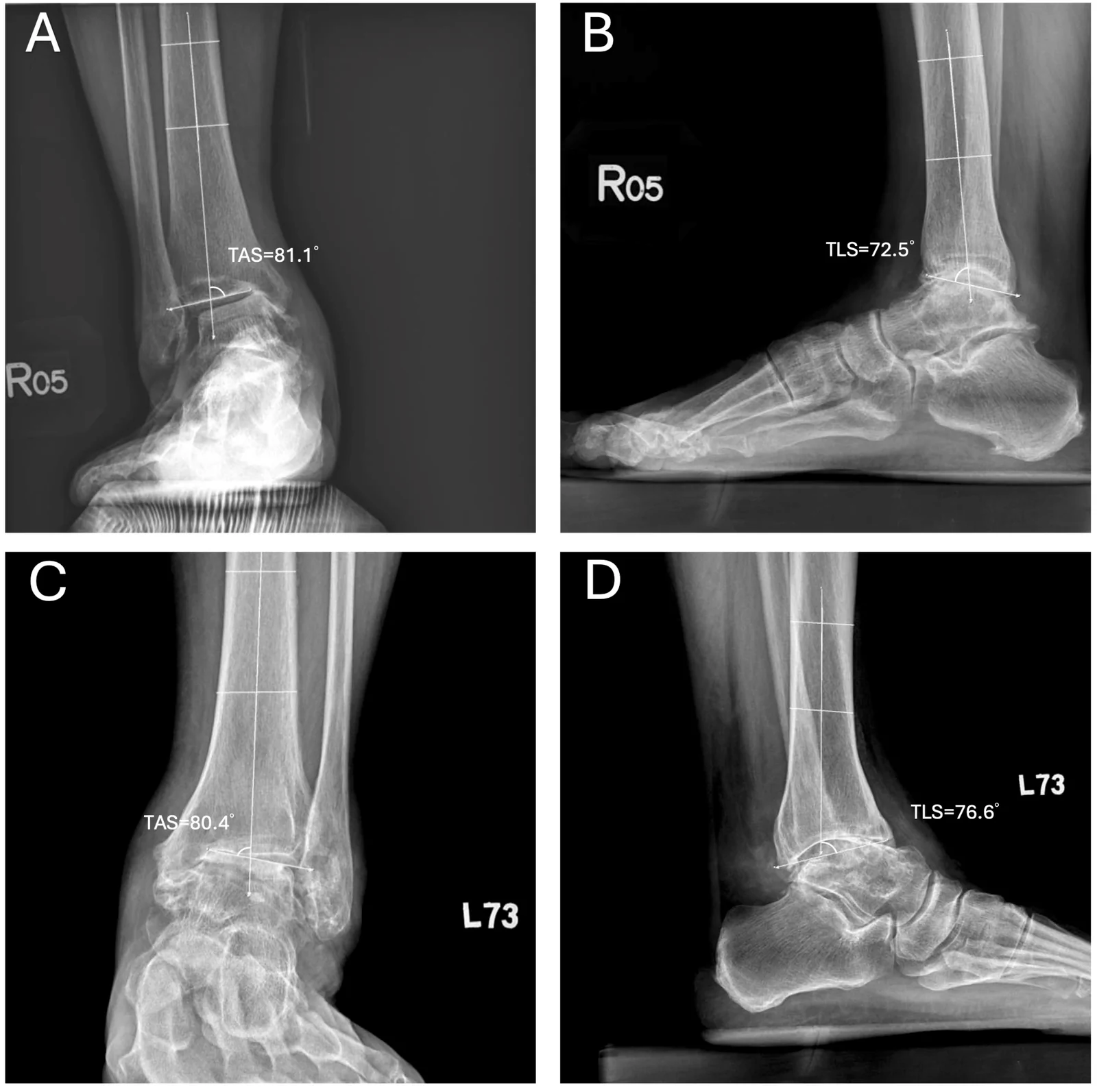
Preoperative weight-bearing radiographs of varus ankle osteoarthritis (Takakura stage IIIa and IIIb). (**A**) (Stage IIIa) Preoperative AP view demonstrates varus ankle OA with subchondral sclerosis and joint space narrowing, predominantly in the medial gutter. TAS 81.1°, MMA 40.1°, TTA 2.9°, TTS 77.2°. (**B**) (Stage IIIa) Preoperative lateral view reveals osteophyte formation. TLS 72.5°. (**C**) (Stage IIIb) Preoperative AP view demonstrates varus ankle OA with subchondral sclerosis and joint space narrowing, predominantly in the medial gutter, dome. TAS 80.4°, MMA 44.9°, TTA 0.3°, TTS 79.5°. (**D**) (Stage IIIb) Preoperative lateral view reveals talar tilt, subluxation, osteophyte formation. TLS 76.6°.

**Figure 4:**
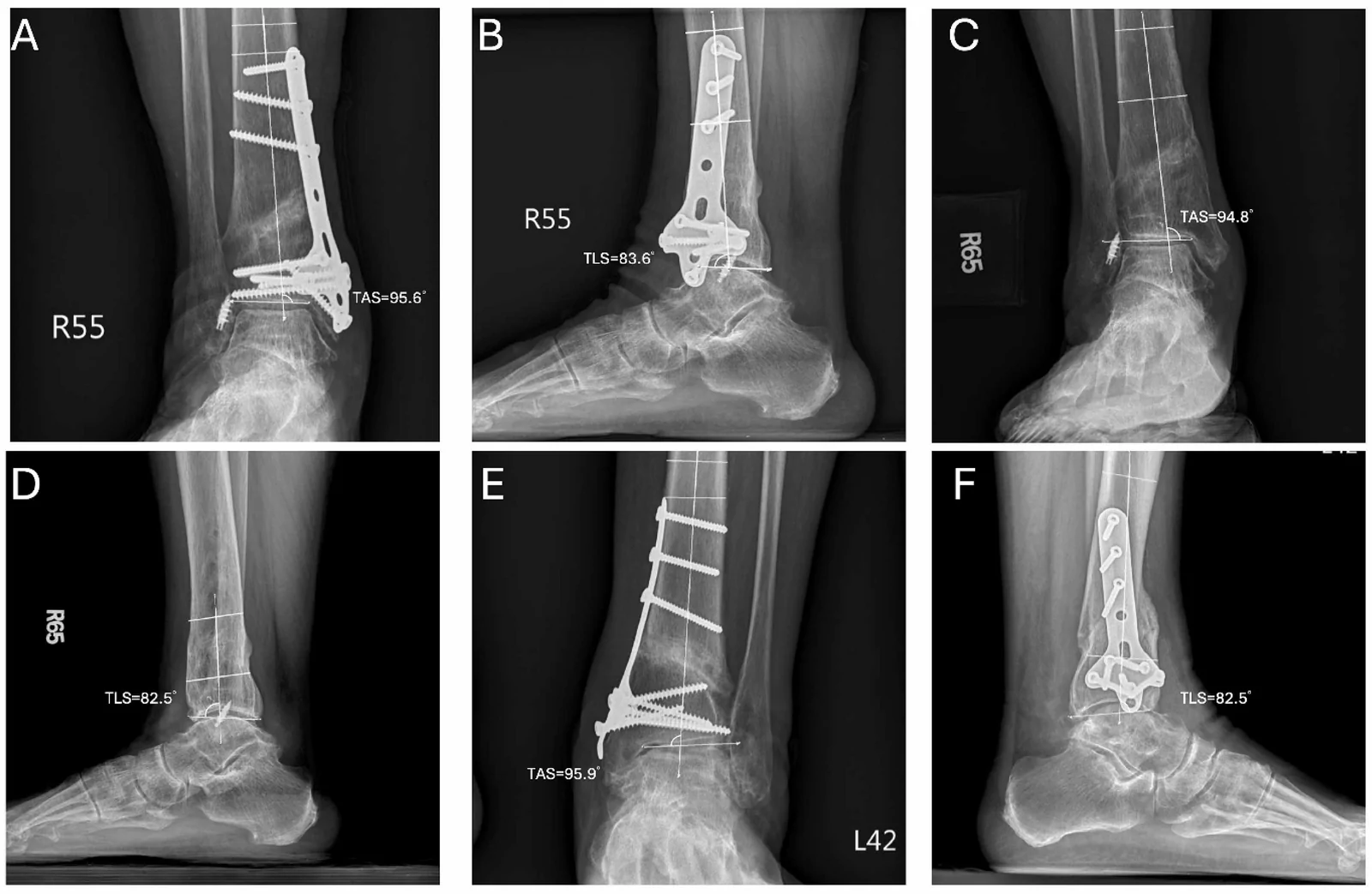
Postoperative weight-bearing radiographs following DTOO. (**A**) (Stage IIIa) Postoperative AP radiograph at 1 year after DTOO with strut allograft insertion and medial plating, ATFL repair additionally. Correction of joint alignment is noted with restoration of the joint line and reduction in the tibiotalar tilt. Allograft incorporation and bony union are observed without hardware failure. TAS 95.6°, MMA 27.7°, TTA 3.9°, TTS 92.3°. (**B**) (Stage IIIa) Postoperative lateral radiograph at 1 year. The anterior impingement spur and dome flattening observed preoperatively have been addressed via realignment. TLS improved to 83.6°, and sagittal plane congruency of the tibiotalar joint is preserved. (**C**) (Stage IIIa) Postoperative AP radiograph at 3 years, 1 year after plate removal. Joint space remains preserved with no recurrence of varus tilt or medial gutter narrowing. The allograft appears fully consolidated without subsidence. No signs of osteophyte formation or sclerosis. TAS 94.8°. (**D**) (Stage IIIa) Postoperative lateral view at 3 years. Sagittal alignment is maintained, with no anterior impingement recurrence. The tibial plafond and talar dome show no progression of arthritic changes. TLS 82.5°. (**E**) (Stage IIIb) Postoperative AP radiograph at 1 year after DTOO with strut allograft insertion and medial plating, ATFL repair additionally. Correction of joint alignment is noted with restoration of the joint line and reduction in the tibiotalar tilt. Allograft incorporation and bony union are observed without hardware failure. TAS 95.9°, MMA 36.3°, TTA 1.4°, TTS 89.3°. (**F**) (Stage IIIb) Postoperative lateral radiograph at 1 year. The anterior impingement spur and dome flattening observed preoperatively have been addressed via realignment. TLS improved to 82.5°, and sagittal plane congruency of the tibiotalar joint is preserved.

Figure 3 and Figure 4 present representative preoperative and postoperative weight-bearing radiographs of patients with Takakura stage IIIa and IIIb disease. These radiographs demonstrate restoration of ankle alignment, correction of medial joint space narrowing, and maintenance of tibiotalar joint congruity at mid-term follow-up.

## 4. Discussion

Varus ankle osteoarthritis involves asymmetric load distribution across the tibiotalar joint, mainly due to medial joint degeneration and coronal plane malalignment. Distal tibial varus deformities disrupt force transmission and create uneven contact stress, leading to cartilage degeneration [15]. Therefore, surgical treatment should focus on mechanical realignment and restoration of joint congruity to normalize load transfer and maintain function. The weight-bearing line (WBL) in a properly aligned ankle passes through the talar dome, ensuring even load distribution [16]. Varus deformities shift the WBL medially, thereby increasing stress on the joint.

The primary objective of realignment osteotomies, including DTOO, is to restore a more central passage of the WBL through the ankle joint. A quantitative radiographic study indicated that SMO can lateralize the WBL in patients with varus ankle osteoarthritis [17]. Another study reported that coronal alignment restoration and load transfer centralization are associated with improved clinical outcomes and delayed degenerative progression [18]. In the present cohort, the postoperative increase in TAS and decrease in MMA reflected correction of distal tibial varus alignment, whereas the increase in TTS indicated improvement in tibiotalar joint orientation and congruity. These radiographic changes were consistent with the intended centralization of load transfer across the ankle, although the WBL was not directly measured. These findings underscore the importance of WBL correction as a fundamental biomechanical objective in joint-preserving osteotomy for patients with varus ankle osteoarthritis [19].

Dome osteotomy enables multiplanar correction around a rotational center close to the ankle joint, which facilitates 3-dimensional deformity correction without requiring structural graft interposition [20]. However, this technique may not provide the same structural support achieved by wedge-based reconstruction, particularly in cases requiring controlled angular opening and mechanical support [21].

Our approach employs a strut allograft with an adjustable anterior–posterior height, which enables angular correction and volumetric modulation of the osteotomy gap. This configuration may allow controlled realignment while minimizing excessive medial cortex lengthening and avoiding overdistraction of surrounding soft tissues. Anterior or posterior graft contouring permits individualized gap balancing according to the deformity’s rotational profile while preserving the structural stability of wedge-based reconstruction. Although further comparative investigation is warranted, the findings of this case series suggest that shape-adjustable allografts may be a feasible option for selected patients with rotation-dominant deformities.

Autologous tricortical iliac crest grafts have long been regarded as the gold standard for osteotomies because of their osteoconductive and osteoinductive properties, with consistently high union rates reported in the literature [22]. However, donor-site morbidity remains a major concern [23].

Utilizing a prefabricated β-TCP wedge in open-wedge high tibial osteotomy reduces donor-site complications but is limited to osteoconduction. According to the literature, β-TCP with 60% porosity tends to resorb slowly, leaving residual material that can delay bone union. Variants with higher porosity (75%) are resorbed faster but have lower compressive strength, which may lead to collapse under load [24]. Bone substitute fractures occur in up to 50% of cases within 1 month, potentially leading to loss of correction despite mitigating the risk of screw breakage [25]. Utilizing granular β-TCP leads to higher nonunion rates for gaps under 10° compared with the no-filler treatment. Open-wedge high tibial osteotomy is associated with an increased risk of delayed healing and lateral hinge fractures, which may increase with poor β-TCP resorption [26].

Structural allografts may be useful when autograft harvesting is not preferred. In the present study, fresh-frozen allografts were used, which have been shown to exert osteoconductive and potential osteoinductive effects [27]. The routine use of structural allografts at our institution is supported by an established bone bank with a reliable supply of anatomically matched structural allografts obtained through body donation. These grafts avoid iliac-crest harvesting, provide immediate structural support, and can be individually fashioned to match the open-wedge space. All patients achieved radiographic union within 3 months.

In the present study, solid bone union was observed in all patients. No graft collapse, loss of correction, revision grafting, conversion to ankle arthrodesis, or conversion to total ankle arthroplasty was observed during follow-up. Two patients underwent elective implant removal after confirmed union because of symptomatic hardware irritation or foreign-body sensation. These findings are consistent with those of Myerson et al. [28], who reported a union rate of 92% after complex hindfoot reconstruction procedures involving similar grafts, and with the low complication rates reported by Ahn et al. [29] after distal tibial osteotomy without fibular osteotomy. Additional comparative trials involving serial computed tomography imaging may further elucidate the decision-making process for choosing between β-TCP and allografts, particularly regarding bone union rate, graft remodeling, and long-term alignment maintenance.

In this study, both stage IIIa and IIIb patients demonstrated postoperative improvement in clinical outcomes. All MOXFQ domains and the total index showed major postoperative improvements, with scores nearing the lower end of the scale. The improvements exceeded the minimal clinically important difference of approximately 10–15 points, supporting the clinical relevance of the observed changes. The clustering of scores at the lower end suggests a floor effect, which may have limited the detection of between-group differences at final follow-up. This pattern was consistent across all MOXFQ domains and was supported by parallel improvements in AOFAS, VAS, and SF-12 scores. Although stage IIIb patients had more advanced preoperative disease, no significantly worse postoperative patient-reported outcomes were observed in this subgroup. However, because the subgroup analysis was exploratory and underpowered, these findings should be interpreted descriptively rather than as evidence of equivalent efficacy between stage IIIa and IIIb disease.

DTOO may be particularly useful in selected patients with severe varus ankle osteoarthritis requiring correction of substantial coronal malalignment. The choice among joint-preserving osteotomy, ankle arthrodesis, and total ankle replacement should be individualized according to residual joint preservation, deformity correctability, bone quality, soft-tissue balance, and patient expectations. Arthrodesis provides reliable pain relief but sacrifices tibiotalar motion, whereas total ankle replacement preserves motion but may be less suitable in the presence of substantial uncorrected deformity, compromised bone stock, or soft-tissue imbalance [9,10]. In contrast, DTOO addresses the underlying malalignment while retaining the native ankle joint and may therefore be most appropriate in patients with correctable varus deformity and sufficient residual joint preservation. A contemporary systematic review of SMO reported favorable mid-term outcomes; however, it also indicated a 28% reoperation rate and cases of correction loss [30]. These findings highlight the importance of maintaining correction in severe malalignment. In the present study, radiographic correction was maintained during follow-up, suggesting that DTOO combined with structural strut allografting may provide a stable joint-preserving option in carefully selected patients. However, unlike arthrodesis or total ankle replacement, the durability of DTOO depends on maintenance of correction and the condition of the remaining articular cartilage.

Taken together, DTOO combined with structural strut allografting may preserve ankle motion while allowing individualized angular and rotational correction, immediate structural support of the opening wedge, and avoidance of autologous graft harvesting. Potential disadvantages include the technical demands of individualized graft preparation, dependence on access to appropriately sized structural allografts, and limited comparative evidence regarding its superiority over alternative graft materials or realignment techniques. From a practical perspective, orthopedic surgeons may consider DTOO combined with structural strut allografting as a joint-preserving option for carefully selected patients with Takakura stage IIIa or IIIb varus ankle osteoarthritis. Radiographic follow-up should include serial weight-bearing assessment of ankle alignment, graft incorporation, maintenance of correction, and implant stability using parameters such as TAS, TLS, MMA, TTA, and TTS. Rehabilitation professionals should coordinate progression of weight bearing with clinical tolerance and radiographic healing, beginning with partial weight bearing at 4–6 weeks and advancing toward full weight bearing at approximately 3 months.

However, this study has several limitations. First, its retrospective design, relatively small sample size, and lack of a control group limit the strength of the conclusions and the generalizability of the findings. Second, the exploratory subgroup analysis may have been underpowered, and postoperative floor effects in MOXFQ scores may have reduced our ability to detect between-group differences. Third, multiple subgroup comparisons were performed without adjustment for multiple testing, increasing the risk of type I error. Finally, because concomitant arthroscopic procedures and ligament repair were performed when clinically indicated, these adjunctive procedures may also have contributed to postoperative symptom improvement. Therefore, the clinical outcomes observed in this study should be interpreted as the overall effect of the comprehensive joint-preserving surgical strategy rather than DTOO alone. Larger prospective comparative studies with longer follow up are warranted to further validate the effectiveness and safety of DTOO combined with structural strut allografting.

## 5. Conclusions

In this retrospective case series, DTOO with structural strut allografting was associated with clinical improvement, alignment correction, and reliable bone union in carefully selected patients with Takakura stage IIIa or IIIb varus ankle osteoarthritis. No major complications were observed, and 2 patients underwent elective implant removal after confirmed union because of symptomatic hardware irritation or foreign-body sensation. To corroborate these findings and explore the additional potential benefits of DTOO combined with strut allografting, additional studies should be conducted with larger sample sizes, longer follow-up periods, and comparative study designs. By continually refining and optimizing DTOO, orthopedic surgeons may expand treatment options for patients with complex ankle osteoarthritis, thereby improving their quality of life and functional outcomes.

## Figures and Tables

**Table 1:** Patient demographics and baseline characteristics.

Patient	Age at Surgery (y)	Gender	Side	Takakura Stage	BMI	Follow-Up Period (mo)
1	59	F	R	IIIa	31.7	58
2	62	F	L	IIIa	35.2	52
3	60	M	R	IIIb	28.1	41
4	63	M	R	IIIa	31.5	95
5	67	F	R	IIIa	20.5	69
6	62	F	R	IIIa	23.1	39
7	65	F	L	IIIa	27.0	67
8	64	F	R	IIIa	29.5	45
9	62	M	L	IIIb	23.4	48
10	51	F	L	IIIb	24.7	130
11	50	F	L	IIIb	22.6	146
12	61	F	R	IIIb	33.5	24
13	57	F	R	IIIb	26.0	32
14	63	M	L	IIIb	31.2	24
15	57	F	R	IIIa	26.1	30
16	42	M	L	IIIa	24.5	24
17	64	F	R	IIIa	25.2	24
18	61	M	R	IIIa	26.6	28
19	58	M	R	IIIa	28.9	28
20	59	F	R	IIIa	23.7	24

Cohort summary: right ankle, 13 patients (65%); left ankle, 7 patients (35%); Takakura–Tanaka stage IIIa, 13 patients (65%); stage IIIb, 7 patients (35%).

**Table 2:** Comparison of Preoperative and Postoperative Clinical Outcomes (Wilcoxon signed-rank).

Clinical Outcomes	Preoperative	Postoperative	*p* Value
**Total MOXFQ score**	57.66 ± 22.01	10.63 ± 9.72	<0.001 *
Pain	47.50 ± 26.58	7.50 ± 8.96	<0.001 *
Walking/Standing	71.07 ± 24.58	15.00 ± 14.41	<0.001 *
Social Interaction	46.88 ± 29.56	6.88 ± 8.58	<0.001 *
**Total AOFAS score**	63.75 ± 15.17	85.45 ± 8.37	<0.001 *
AOFAS pain	21.50 ± 12.26	35.00 ± 6.07	<0.001 *
AOFAS function	33.55 ± 10.07	40.45 ± 6.07	0.02 *
AOFAS alignment	8.70 ± 1.17	10.00 ± 0.00	<0.001 *
**SF-12 PCS**	45.23 ± 8.48	47.96 ± 7.93	0.258
**SF-12 MCS**	53.51 ± 9.85	55.60 ± 4.75	0.377
**VAS score**	4.30 ± 1.75	1.00 ± 1.30	<0.001 *

* Statistically significant at *p* < 0.05. Data are reported as mean ± standard deviation. Abbreviations: MOXFQ, The Manchester–Oxford Foot Questionnaire; AOFAS, American Orthopaedic Foot and Ankle Society; SF-12, 12-Item Short-Form Health Survey; VAS, visual analog scale. The AOFAS ankle-hindfoot score comprises pain (0–40 points), function (0–50 points), and alignment (0–10 points), with a total score ranging from 0 to 100 points. Values in bold indicate statistically significant differences (*p* < 0.05).

**Table 3:** Comparison of Preoperative and Postoperative Radiological Outcomes (paired *t*-test).

Radiological Outcomes	Preoperative	Postoperative	*p* Value
**TAS**	83.13 ± 4.88	92.88 ± 4.34	<0.001 *
**TLS**	77.76± 5.60	82.94 ± 8.01	<0.001 *
**MMA**	35.93 ± 7.31	30.05 ± 8.36	0.019 *
**TTA**	5.80 ± 6.70	4.29 ± 3.68	0.336
**TTS**	78.22 ± 6.87	87.79 ± 8.51	<0.001 *

* Statistically significant at *p* < 0.05. Data are reported as mean ± standard deviation. Abbreviations: TAS, Tibial anterior surface angle; TLS, Tibial lateral surface angle; MMA, Medial malleolar angle; TTA, Talar tilt angle; TTS, Tibiotalar surface angle. Values in bold indicate statistically significant differences (*p* < 0.05).

**Table 4:** Exploratory subgroup analysis by preoperative stage (Mann–Whitney U test (two-sided)).

Outcome (Δ, Improvement)	Stage IIIa (*n* = 13)	Stage IIIb (*n* = 7)	*p* Value (Δ)
**Clinical Outcomes**			
Total MOXFQ score	−44.2 ± 22.1	−46.3 ± 21.4	0.692
Total AOFAS score	+22.8 ± 14.9	+24.9 ± 11.2	0.204
**SF-12 PCS**	+1.64 ± 12.33	+4.75 ± 5.92	0.536
**SF-12 MCS**	+3.02 ± 12.54	+0.37 ± 4.25	0.817
VAS score	−3.5 ± 2.1	−3.0 ± 1.7	0.683
**Radiological Outcomes**			
TAS	+9.7 ± 4.6	+9.9 ± 5.1	0.438
TLS	+5.6 ± 6.4	+4.3 ± 7.1	0.183
MMA	−5.6 ± 6.8	−6.4 ± 8.9	0.757
TTA	−1.4 ± 2.2	−4.8 ± 5.6	0.024 *
TTS	+8.9 ± 6.1	+10.6 ± 8.9	0.135

* Statistically significant at *p* < 0.05. Data are reported as mean ± standard deviation. Abbreviations: MOXFQ, The Manchester–Oxford Foot Questionnaire; AOFAS, American Orthopaedic Foot and Ankle Society; SF-12, 12-Item Short-Form Health Survey; VAS, visual analog scale. TAS, Tibial anterior surface angle; TLS, Tibial lateral surface angle; MMA, Medial malleolar angle; TTA, Talar tilt angle; TTS, Tibiotalar surface angle. Values in bold indicate statistically significant differences (*p* < 0.05).

## Data Availability

The data supporting the findings of this study are available from the corresponding author upon reasonable request. The data is not publicly available due to privacy and ethical restrictions.

## Abbreviations

The following abbreviations are used in this manuscript:

AOFAS	American Orthopaedic Foot & Ankle Society
AP	Anteroposterior
ATFL	Anterior talofibular ligament
β-TCP	Beta-tricalcium phosphate
DTOO	Distal tibial oblique osteotomy
IRB	Institutional Review Board
MMA	Medial malleolar angle
MOXFQ	Manchester–Oxford Foot Questionnaire
OA	Osteoarthritis
SF-12	12-Item Short-Form Survey
SMO	Supramalleolar osteotomy
TAS	Tibial articular surface angle
TLS	Tibial lateral surface angle
TTA	Talar tilt angle
TTS	Tibiotalar surface angle
VAS	Visual Analog Scale
WBL	Weight-bearing line
